# Analysis of Food Insecurity in U.S. Colleges Using Current Assessment Tools—A Systematic Review

**DOI:** 10.3390/nu18121866

**Published:** 2026-06-10

**Authors:** Qi Fu, Maggie Cappiello, Elizabeth M. Gardner

**Affiliations:** 1 Department of Food Science and Human Nutrition, Michigan State University, East Lansing, MI 48823, USA; fuqi@msu.edu (Q.F.); cappiel5@msu.edu (M.C.); 2 Department of Pharmacology and Toxicology, Michigan State University, East Lansing, MI 48823, USA

**Keywords:** food insecurity, college students, surveys and questionnaires, United States, systematic review

## Abstract

**Objectives**: Food insecurity (FI) among college students is an emerging global public health concern. While the burden is international in scope, this systematic review evaluates the prevalence of FI in college populations in the United States (U.S.) and examines the suitability of commonly used FI assessment tools for this population. **Methods**: A systematic search of PubMed, Scopus, and Web of Science was conducted (up to April 2026) in accordance with the PRISMA 2020 Abstracts checklist. Eligible studies were peer-reviewed research articles published between 2005 and 2026, conducted in the U.S., written in English, and including college or university students with sample sizes ≥ 30. Studies were required to use validated FI assessment tools developed by the United States Department of Agriculture (USDA) or Health Watch. Study quality was assessed using the Joanna Briggs Institute Critical Appraisal tools and only studies rated as moderate or high quality were included. Results were synthesized by grouping studies according to the FI assessment tools used. **Results**: Thirty studies met the inclusion criteria (total *n* = 213,624 students surveyed). FI prevalence among U.S. college students ranged from 14% to 72.9%. Variability in estimates was influenced by the assessment tool used, demographic characteristics, institutional settings, and regional socioeconomic differences. Shorter screening instruments, including the USDA six-Item Household Food Security Survey Module (HFSSM) Short Form and Hunger Vital Sign, demonstrated greater variability in reported FI prevalence (47% and 41%, respectively) compared with longer assessment measures. Higher FI prevalence was also more frequently reported among students of color, those from lower socioeconomic backgrounds, and female students. **Conclusions**: Findings demonstrate FI is prevalent among college students. Limitations of the current study include restriction to three databases, exclusion of pre-2005 studies, and inclusion of only U.S.-based studies. Variability in assessment methods, as well as consideration of confounding variables (e.g., socioeconomics, demographics and institutional settings), underscores the need for context-specific tools tailored to this population to inform effective interventions and policies globally.

## 1. Introduction

Food insecurity (FI) has been a significant public health concern for decades, and the COVID-19 pandemic further intensified attention to the issue globally. Post-pandemic, over 2.3 billion people experience moderate or severe FI worldwide in 2023, a level that remains persistently elevated compared with pre-pandemic estimates [1]. Even in the United States (U.S.), a high-income country, approximately 12.8% households reported experiencing FI in 2022, and the percentage rose to 13.5% in 2023, reflecting a continued increase in severity [2]. While food security remains a major concern at the household level, it is increasingly recognized among college students, a rapidly growing population across the globe [3]. In the U.S., over 19 million individuals were enrolled in post-secondary institutions in 2022 [4]. Recent evidence has also highlighted the severity of FI in higher education across multiple other countries, including Australia, Brazil, Bangladesh, Canada, German, Iceland, Jordan, Malaysia, Nigeria, Saudi Arabia, and the United Kingdom [4,5,6,7,8,9].

Several tools are used to assess FI in the U.S. According to the United States Department of Agriculture (USDA), food security is defined as consistent access to adequate food and is categorized into four levels: high, marginal, low, and very low [10]. Low and very low food security are classified as FI, defined as “limited or uncertain access to adequate food” [10]. Very low food security, often referred to as hunger, is characterized by disrupted eating patterns and reduced food intake [10]. To assess FI, there are four main validated instruments used in the U.S., primarily developed by the USDA and Health Watch, each of which differ in length and level of detail. The full USDA Household Food Security Survey Module (or HFSSM; 18-item) is the most comprehensive tool, capturing experiences like anxiety about food access, reduced diet quality, and disrupted eating patterns [11]. The short USDA Household Food Security Survey Module (or HFSSM Short Form; six-item) is an abbreviated version that focuses on more severe, behavior-based indicators of FI [11]. The Adult Food Security Survey Module (or AFSSM; 10-item) is simpler compared with the full HFSSM and captures both anxiety-related and behavioral components of FI, though with less granularity [11]. In contrast, the Health Watch Vital Sign (HVSS) survey is a brief two-item screening tool used for rapid identification of FI risk, particularly in clinical settings, yet it does not assess severity [12].

Although these tools were originally designed for household-level assessment, due to the lack of student-specific instruments, they are frequently applied to college populations because they are generally self-administered, impose minimal participant burden, and are widely accessible online. However, given the unique characteristics of college students, such as independent living arrangements, housing instability, and variable access to campus food resources, these existing tools may not adequately capture FI in this population. Thus, the purpose of this review is to (1) examine the severity of FI among U.S. college students and (2) evaluate current assessment tools used in the U.S. to inform potential improvements for use in this population.

## 2. Materials and Methods

### 2.1. Eligibility Criteria

Study selection criteria included: (a) publication dates between 1 January 2005 and 30 April 2026 to focus on the most recent data; (b) study location in the U.S.; (c) publication in English due to translation limitations; (d) research articles only; (e) availability of full articles; (f) adequate sample size (*n* ≥ 30); (g) study subjects enrolled in a college or university; (h) use of USDA or Health Watch measurement tools due to established validity, standardized use, and comparability across studies. Full-text availability was restricted directly at the database level during the initial search phase using built-in search engine filters. This automated filtering step excluded non-article records, such websites, abstracts, news, documentation, book reviews, editorials, conference proceedings, letters to the editor, and patent filings. Included studies were grouped for synthesis according to the FI measurement tool used (e.g., USDA full HFSSM, USDA short HFSSM, AFSSM, and HVSS).

### 2.2. Search Strategy

This systematic review followed the guidelines of the Preferred Reporting Items for Systematic Reviews and Meta-Analysis (PRISMA) 2020. A systematic search was conducted in PubMed, Scopus, and Web of Science (WoS) databases. The final search was completed in May 2026. Key search terms used in PubMed included: (“Food Insecurity”[Mesh] OR “food insecurity”[tiab] OR “Food Security”[Mesh] OR “food security”[tiab] OR “Hunger”[Mesh] OR hunger[tiab]) AND (“Students”[Mesh] OR “Universities”[Mesh] OR “Education, Graduate”[Mesh] OR “Academia”[Mesh] OR college[tiab] OR university[tiab] OR undergraduate*[tiab] OR college student*[tiab] OR university student*[tiab]) AND (“United States”[Mesh] OR U.S.[tiab] OR USA[tiab] OR America[tiab]). Key search terms used in Scopus included: TITLE-ABS-KEY ((“food insecurity” OR “food security” OR hunger) AND (“college student*” OR “university student*” OR college OR university OR undergraduate) AND (“United States” OR USA)). Key search terms used in WoS included: “food insecurity” OR hunger AND (college OR university OR undergraduate students) (Title).

The complete search strategies for all databases and the PRISMA 2020 Checklist for reproducibility in systematic reviews are provided in Supplementary Materials.

### 2.3. Study Selection and Data Synthesis

After study selection based on eligibility criteria, studies retrieved from database searches were manually screened for relevance. Study selection was initially conducted by two reviewers (Q.F. and E.M.G.), with eligibility decisions confirmed through consensus among the reviewers.

Standardized risk of bias was assessed independently by two reviewers (Q.F. and E.M.G) using the Joanna Briggs Institute (JBI) Critical Appraisal Checklist (Supplementary Materials Table S1) [13]. Studies were categorized as high, moderate, or low quality based on the number of criteria fulfilled, and only studies rated as high or moderate quality were included.

Data were then extracted from each included study by the above two reviewers independently and organized into a standardized summary table, with the following headings: (1) measurement tool; (2) characteristics of the tool; (3) number of studies using the tool; (4) number of students surveyed by author; (5) FI rate by author; and (6) reported range of FI prevalence (%)No study investigators were contacted for additional data. Due to variability in study populations and measurement instruments, a meta-analysis was not performed. Instead, a narrative synthesis was conducted. Studies were also grouped according to FI assessment tool, and results were compared descriptively across instruments and population subgroups.

## 3. Results

**Figure 1:**
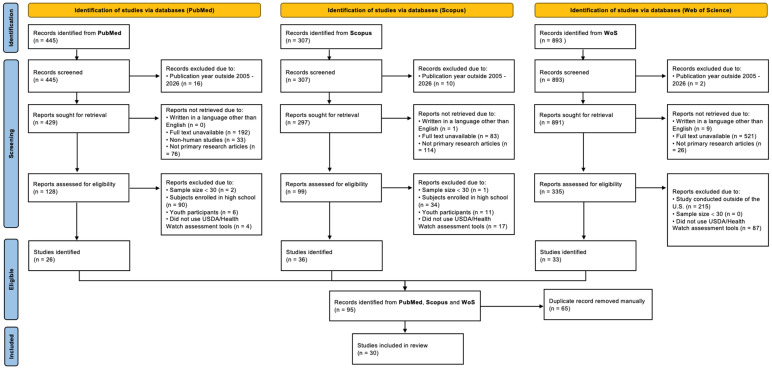
PRISMA 2020 flow diagram for record identification and selection for a systematic review of the severity of food insecurity among college students in the United States.

The initial keyword search identified 445 records from PubMed, 307 records from Scopus, and 893 records from WoS. After removal of studies published outside the 2005–2026 range, 429 PubMed records, 297 Scopus records, and 891 WoS records were retained for screening. During the full-text retrieval phase, PubMed records were excluded due to full-text unavailability (e.g., websites, abstracts, news, documentation, book reviews, editorials, conference proceedings, letters to the editor, and patent filings *n* = 192), non-human studies (*n* = 33), and non-primary research articles (*n* = 76). Scopus records were excluded due to non-English language (*n* = 1), full-text unavailability (e.g., websites, abstracts, news, documentation, book reviews, editorials, conference proceedings, letters to the editor, and patent filings, *n* = 83), and non-primary research articles (*n* = 114). WoS records were excluded due to non-English language (*n* = 9), full-text unavailability (e.g., websites, abstracts, news, documentation, book reviews, editorials, conference proceedings, letters to the editor, and patent filings (*n* = 26). This resulted in 128 PubMed, 99 Scopus, and 335 WoS records being assessed for eligibility. Studies were then excluded for sample size ≤ 30 (PubMed *n* = 2; Scopus *n* = 1; WoS = 0), enrollment of high school participants (PubMed *n* = 90; Scopus *n* = 34), inclusion of youth participants (PubMed *n* = 6; Scopus *n* = 11), studies conducted outside of the U.S. (WoS = 215), and used tools other than the USDA or Health Watch FI assessments (PubMed *n* = 4; Scopus *n* = 17; WoS = 87). This yielded 26 eligible PubMed studies, 36 eligible Scopus studies, and 33 eligible WoS studies. A total of 95 studies were identified across all databases. After manual removal of duplicates (*n* = 65), 30 studies were included in the final analysis of this review, comprising a total of 213,624 participants (Figure 1). Results were synthesized according to the four FI assessment tools used across the included studies (Table 1).

### 3.1. USDA Household Food Security Survey Module

Four studies employed the USDA Household Food Security Survey to measure FI among undergraduate students (*n* = 4483) [14,15,16,17]. Three of the four were cross-sectional in design. Payne-Sturges et al. reported a 15% FI rate in a cross-sectional study of 237 students at a Mid-Atlantic university [14]. An additional 16% of participants were identified as at risk of hunger, with off-campus and marginalized students being more vulnerable than their White, on-campus peers [14]. Similarly, Wolfson et al. found an average of 15% FI rate among 2538 students surveyed from multiple public universities and community colleges between 2015 and 2019 [15]. This study also found that Black and Hispanic students were more likely to experience FI compared with White students, and that first-generation students and those who were financially independent from their parents, had higher rates of FI [15].

In contrast, a 2017 cross-sectional study of 217 community college students across 12 Maryland institutions, found that 52% of participants experienced FI while enrolled; a much higher rate compared with the previous two studies [16]. Notably, women represented 59% of the study population, with single parents and women of color experiencing the highest levels of FI [16]. Odds ratios (OR) further indicated a higher risk among students from low-income households (Pell Grant recipients) (OR = 2.3) and women over the age of 20 (OR = 2.8) compared with non-recipients and younger women [16].

Leung et al. conducted a longitudinal study assessing FI rates during college enrollment (1999–2003) and again in adulthood (2015–2017) [17]. Among 1508 students, the baseline prevalence of FI during college was 14.9% [17]. Students who were non-White, had lower parental education, and were economically independent were more likely to be food insecure [17]. Importantly, FI during college was associated with a 45% greater likelihood of experiencing hunger in adulthood, highlighting the long-term implications of FI in this population [15]. Overall, most studies reported FI prevalence of approximately 15%, although higher estimates were observed in samples with a greater proportion of female and single parent participants [14,15,16,17].

### 3.2. USDA Adult Food Security Survey Module

Eight cross-sectional studies applied the USDA Adult Food Security Survey to assess FI on U.S. college campuses (*n* = 19,049) [18,19,20,21,22,23,24,25]. The findings of these studies consistently demonstrated substantial variability in prevalence, ranging from approximately 14% to over 40%, depending on institutions and student demographics.

Chaparro et al. studied 441 students at the University of Hawaii Manoa and found a 21% FI rate, with an additional 24% identified as at risk [25]. Notably, those living off-campus, and those identifying as Hawaiian, Filipino, and Pacific Islander, were disproportionately affected [25]. Similarly, Batchelder et al. reported a comparable overall prevalence of 22% across 4140 students from private universities, Historically Black Colleges and Universities (HBCUs), and community colleges in the southeastern U.S. However, marked disparities emerged across institution types, with FI affecting 37.1% of HBCU students and 29.2% of community college students, compared with 11.2% in private university students [19].

El Zein et al. further highlighted that both geographic and socioeconomic variations impact food security. In their 2019 multi-university study (*n* = 855), FI prevalence averaged 19.1%, ranging from 7.1% to 25% across campuses [20]. A 2020 follow-up study across eight additional universities (*n* = 683) reported similar variability (19–24.1%) and identified higher odds of FI among Pell Grant recipients (OR = 1.95), employed students (OR = 1.6), and off-campus residents (OR = 1.99) [21]. Moreover, Fiagbor et al. assessed 462 students from three public U.S. universities and reported 14% were food insecure, while 12% were marginally food secure [23]. These rates were relatively lower than those reported in other studies. These lower rates may reflect that the study population consisted predominantly of female (76%) and Caucasian (76%) participants, with most students reporting financial support from family and friends [23]. Further, qualitative interviews (*n* = 26) revealed that students frequently misinterpreted key terms in the USDA AFSSM module [23].

At the single-institution level, Brescia and Cuite reported a notably higher overall prevalence of 41.1% FI among 4491 undergraduate students at Rutgers University, with 18.24% experiencing low food security and 22.91% very low food security [22]. Risk was also patterned by student characteristics, with full-time students being 2.6 times more likely to experience FI, and Hispanic students being 1.6 times more likely than their White peers [22]. Wooten et al. reported a similar 36% FI rate after surveying 4824 students across a large public university system in Southeastern U.S. [18]. In contrast, Glick et al. surveyed 853 students at a Midwestern U.S. university and reported a lower FI prevalence of 26.7% (14.4% low, 12.3% very low food security) [24]. However, the authors also noted that FI prevalence differed significantly across demographic groups, with higher rates observed among international students (26.8%) and U.S. multicultural students (35.6%) compared with White students (19.9%; *p* < 0.001) [24].

### 3.3. USDA Household Food Security Survey Module (Short Form)

The USDA HFSSM six-item survey was utilized in 14 studies, making it the most common tool employed in this review (*n* = 62,373) [26,27,28,29,30,31,32,33,34,35,36,37,38,39]. Thirteen studies employed a cross-sectional design [26,27,28,29,30,31,32,33,34,35,37,38,39]. For comparison among studies, we have grouped the studies by percentage of FI reported (ranging from highest to lowest). The highest reported prevalence was 59–61%, observed by Patton-Lopez et al. among 354 students at Western University [26] and Zuo et al. among 335 students at a Southwestern U.S. public university (30% low and 31% very low FI) [38]. Regression analysis showed different predictors of food security and cultural food access by students’ region of origin, caregiver status, housing, and vehicle ownership [38].

The second set of studies reported FI rates ranging from approximately 40% to 30%. For example, Marmolejo et al. found a 40.7% FI rate among 48,103 students across 75 U.S. universities [27]. In accordance, Martinez et al. reported an overall 40% FI rate among 8705 college students at University of California, with Hispanic and Asian students facing higher risks of FI (31%) [28], while Becerra MB and Becerra BJ found 37.5% FI among 302 students in a college; interestingly, the survey indicated that 67.9% of students were Hispanic [29]. Reeder et al. reported a slightly low rate of 32.8% FI among 131 students at Mississippi State University, with Black students having 3.5 times higher odds of experiencing hunger compared with their White counterparts [30]. Similarly, Tripathy et al. reported a 32% prevalence among 418 students at Large Public Southeastern University (LPSU), with 60% of Black students and 47.5% of Hispanic students experiencing FI [31]. Additionally, Harville et al. reported a similar 36% prevalence among 1087 students at the University of Florida, with higher risk among those with prior participation in public assistance programs, such as the Supplemental Nutrition Assistance Program (SNAP), Special Supplemental Nutrition Program for Women, Infants, and Children (WIC), or free/reduced-price lunch, and those who reported being financially independent from parents or guardians [32]. And lastly, Cuy Castellanos et al. surveyed 560 undergraduate and graduate students at a mid-size private Catholic university and reported 35.8% of students were FI [39]. Additional logistic analysis showed students prioritizing spending on alcohol or tuition had significantly higher odds of FI [39].

The final group of studies reported lower prevalence estimates, ranging from 25% to 14%. Davidson et al. reported 25% FI prevalence in a survey of 943 university students in New Hampshire [37]. The investigators also found students, who received financial aid and identified as first generation college students, were more likely to be FI [37]. Mobley et al. reported a 23.7% FI rate among the 372 participants at Clemson University, with both non-White and first-generation college students having a significantly higher risk of FI [33]. Participants who experienced hunger during childhood were three times more likely to report current FI compared with students who grew up in food-secure households. Similarly, Fortin et al. found a 23% FI rate among 30 students at the University of Kansas [34]; those who identified as food insecure cited a lack of, not only adequate calories, but also dietary diversity. The lowest estimate was reported by Mei et al., who found a 14% prevalence among 1033, who were primarily early-year students recruited from campus dining halls. Further, FI in this group was, again, more common among first-generation, non-White, and Pell Grant recipient students [35]. Although not a cross-sectional study, it is important to highlight a longitudinal cohort study using this instrument that reported similar FI rates. Raskind et al. indicated a 29% FI rate in 2377 students across seven universities in Georgia surveyed every four months over two years [36].

### 3.4. Health Watch Hunger Vital Sign

Four cross-sectional studies used the online Health Watch Hunger Vital Sign survey to assess FI in college students (*n* = 127,719) [40,41,42,43]. Bruening et al. surveyed 209 first-year college students living on campus at Arizona State University and found that 37% reported FI, defined as inconsistent food access in the previous three months [40]. Similarly, Zickgrif et al. analyzed data between 2020 and 2021 from 121,627 college-aged participants across 140 U.S. colleges and universities and reported a comparable FI prevalence of 31.9% [41].

In contrast, higher rates were observed in studies with different student demographics. Duke et al. surveyed 351 students from four HBCUs in the North, where about 91.2% of participants were Black or African American, and found that 72.9% of students experienced FI over the past 12 months [42]. Likewise, Enriquez and Ader reported that 52% of 3155 students at the University of Tennessee were food insecure [43].

### 3.5. Risk of Bias Assessment

Assessment of bias was performed by following the JBI checklist. Overall, the evidence base is robust: all studies met key JBI criteria (Table 1 and Table S1). Every paper clearly defined its college student population and sampling frame, and described demographics. All used validated instruments for FI. In most cases (28 out of 30), confounders, such as race, gender, year, financial aid, were identified and adjusted for via multivariable logistic or regression analyses [14,15,16,17,18,19,20,21,22,24,25,26,27,28,29,30,31,32,33,35,36,37,38,39,40,41,42,43]. Overall, our review rated 28 studies as high quality and two as moderate quality using the JBI checklist for analytical cross-sectional study designs.

Thus, although findings in this review consistently indicated that FI is prevalent among U.S. college students, variability in measurement tools, participant demographics, recruitment strategies, and the predominance of cross-sectional designs limits the precision and generalizability. In particular, most studies were cross-sectional (29 of 30) and therefore subject to inherent limitations in causal inference [14,15,16,17,18,19,20,21,22,23,24,25,26,27,28,29,30,31,32,33,34,35,37,38,39,40,41,42,43]. Clearly, longitudinal studies would provide additional support for the persistence of FI over time, but such studies are limited in number and in scope. Two studies recruited participants through campus dining or residential halls [35,40]; however, this recruitment approach may introduce selection bias, as students experiencing FI may be less likely to have access to these settings. Studies using multi-institutional samples generally demonstrated lower risk of bias compared with single-institution studies. Additional sources of bias included non-random sampling and variation in recruitment methods, including voluntary survey participation, which may have introduced potential social desirability and self-selection bias, as food-insecure students may be more or less likely to respond.

## 4. Discussion

FI among college students is increasingly recognized as a global public health concern. The transitional nature of university life, including financial instability, limited access to food preparation resources, and shifting in financial support systems, can all contribute to FI in ways that differ from those experienced in a traditional household setting. Thus, this population has become an important focus in recent research. Importantly, this issue is not confined to any single national context. Evidence from multiple countries indicate that college students experience varying degrees of FI [4,5,6,7,8,9]. Within this broader global pattern, findings from the U.S. reflect similar trends. Across the 30 studies included in this review, there was a convergent finding that a substantial proportion of U.S. college students experience FI, though prevalence estimates vary by demographics and the measurement tool used [14,15,16,17,18,19,20,21,22,23,24,25,26,27,28,29,30,31,32,33,34,35,36,37,38,39,40,41,42,43]. Notably, higher rates were more frequently observed among non-White students, first-generation college students, females, individuals with lower parental economic status, and financially independent students [14,15,16,17,18,19,21,22,24,25,27,28,29,30,31,32,33,35,37,38,42].

**Figure 2:**
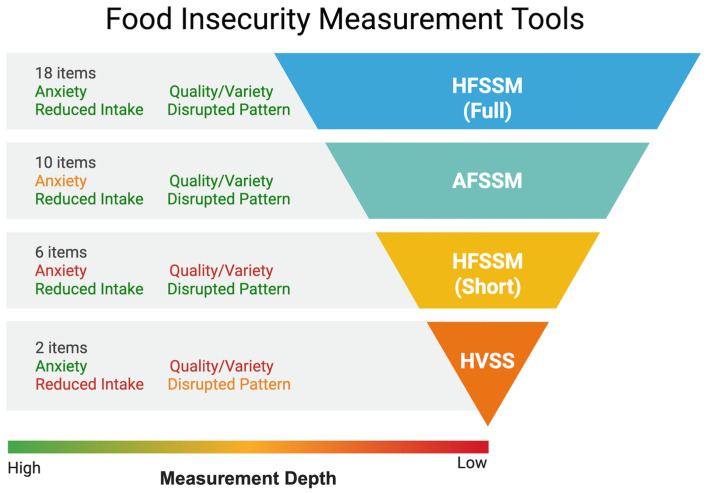
Measurement depth of food insecurity assessment tools—categorized by construct domains. Domains include food-related anxiety, reduced food intake, dietary quality and variety, and disrupted eating patterns. A color gradient is used to represent measurement depth, with red indicating low depth, orange indicating moderate–low depth, and green indicating high depth.

Nearly all studies (26 of 30) assessed FI using standardized USDA instruments, including the full HFSSM (18 items), HFSSM Short Form (six items), and AFSSM (10 items) (Figure 2) [14,15,16,17,18,19,20,21,22,23,24,25,26,27,28,29,30,31,32,33,34,35,36,37,38,39], while the remaining four studies used HVSS (two items), which consists of only two questions [40,41,42,43]. Shorter screening instruments, including the HFSSM Short Form and HVSS, demonstrated greater variability in reported FI prevalence (47 and 41%, respectively), compared with standard-length HFSSM and AFSSM (37.1 and 27.15%, respectively). However, observed differences in prevalence estimates may also be influenced by demographic composition, institutional settings, geographic variation, and underlying socioeconomic conditions. In addition, these prevalence differences may also reflect sex- and gender-related composition effects, as prior work in U.S. college students has identified pregnant and parenting students as emerging high-risk groups, with single parents showing significantly higher odds of FI, financial hardship, and lower academic performance compared with non-parenting peers [44]. Thus, future studies must consider the contribution of these confounding variables when evaluating the various FI assessment tools.

To our knowledge, no validated food security measurement tool currently exists that is designed specifically for college student populations. Although USDA-based tools provide a well-established framework for assessing FI in general populations, they may not fully capture the unique circumstances of university students. Notably, several studies acknowledged the survey instrument itself as a potential source of measurement bias or as having confounding variables. Specifically, the USDA HFSSM and AFSSM do not fully capture the unique food access patterns of college students (Figure 2). For instance, many college students rely on on-campus meal plans, financial support from parents, and experience semester-based changes in housing and food availability. Similarly, the HFSSM Short Form does not adequately reflect the frequency, severity, or anxiety of FI in this group. This instrument also provided limited depth regarding whether students missed meals by choice or due to a lack of resources. In addition, four studies utilized the two-question HVSS to assess FI among college students [40,41,42,43]. Although originally developed for quick clinical screening for households with young children, it has been extrapolated to broader populations due to its low respondent burden. However, its limited item structure restricts the interpretation of severity and context. As noted by Duke et al., the absence of follow-up questions reduces interpretability and limits the ability to distinguish gradations of FI [42], suggesting the need for supplementary measures to improve analytic depth. Collectively, these tools provide useful but incomplete assessments of FI among college students. Hence, the development of an additional USDA survey specifically for college students is warranted to better identify risk within this population (Table 2).

**Figure 3:**
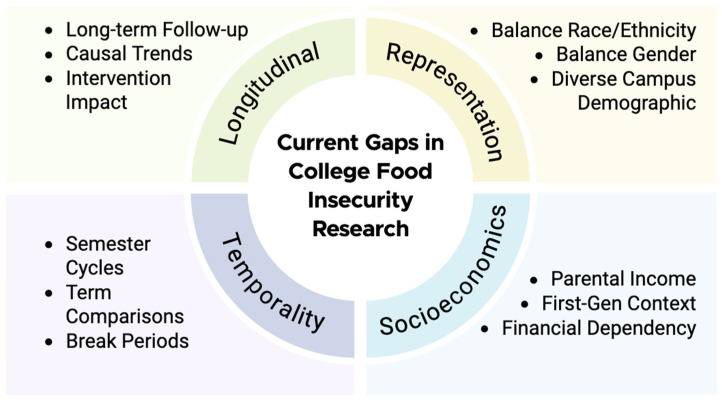
Current gaps in college FI research.

Beyond measurement, several methodological differences in study design (e.g., primarily analytical cross-sectional studies included in this review), contribute to variability in reported FI rates (Figure 3). Importantly, we recognize that high certainty of evidence for analytical cross-sectional studies may not indicate the same high level of confidence seen in studies with more stringent, highly controlled experimental designs, which were not included in this review. In the current review, we have identified several key areas that may weaken the confidence level of the findings in the studies reviewed.

First, limited racial and ethnic diversity may bias estimates and weaken representativeness. Although recruitment is inherently imperfect, efforts should be made to better reflect college student demographics, particularly race and ethnicity. For example, data from HBCUS with over 50% of participants identified as Black or African American showed a distinct higher FI compared with other studies in this review [19,42]. This aligns with broader disparities, as one in three Black people in the U.S. experienced some level of FI, which is largely driven by racism, low wages, high unemployment rates, and living in food deserts [2]. Despite evidence of higher risk profiles, only two of 30 studies focused on HBCUs [19,42], highlighting the need for further research focused on minority college populations.

Along similar lines, unequal gender distribution is a methodological limitation (Figure 3). More than half the included studies were composed of over 60% female students, with some reaching 75–85%. This imbalance partly reflects that women are more likely to participate in surveys compared to men [45]. Despite this, evidence has suggested that women possess a greater risk of facing FI. The study examining the effect of single-parent status on food security found that single mothers were at significantly higher risk of experiencing FI compared to non-parent or married students, highlighting an understudied subgroup within the population [16]. Future studies should aim for more balanced recruitment to better represent all genders across marital statuses and collect data on the number of dependent children, as both may influence FI status. Gender-diverse students should also be included in studies, if possible. According to the Association of American Universities (AAU), 1.7% of college students in the U.S. identified as transgender, nonbinary, or questioning in 2020 [46,47]. Future studies should include a more gender-sensitive approach to FI assessment that considers sex-specific determinants, caregiver responsibilities, socioeconomic status, and intersectional factors. These sex and gender differences clearly influence health outcomes and social determinants of diet [48,49].

Study timing represents another limitation in the included studies (Figure 3). College life takes on a transitional tone; therefore, food security status shifts due to semester breaks. Thus, access to food resources heavily depends on living arrangements and household support when on semester break. As a result, food security status may improve or worsen outside of academic terms. Notably, national data indicate that in 2020, 28.6% of low-income households identified as food insecure compared to the national average of 10.5% [50,51,52,53], underscoring the disproportionate burden faced by economically disadvantaged populations. Prior research also found that food-insecure respondents were more likely to have lived in government-sanctioned housing, used free/reduced price school lunch, used SNAP and/or WIC, received resources from a food bank in the past, and were on financial aid at the time of college enrollment [15,32,36]. Hence, parental socioeconomic status is another essential parameter to consider.

It may be critical that future studies use a prospective cohort design (longitudinal) to provide more comprehensive outlooks of college student FI (Figure 3). This allows studies to (1) establish causation [54]; (2) minimize respondent bias; (3) measure changes in food security over time; and (4) produce more generalizable results for interventions. In addition, more inclusive strategies, such as course-based sampling or university-wide online recruitment, may better capture a representative student population across food security statuses. As recruiting from dining halls or residence halls may introduce selection bias by overrepresenting on-campus students, particularly first-years and those with dining plans, who may be less likely to experience FI.

All articles included in this systematic review were identified through three databases: PubMed, Scopus, and Web of Science. While all databases are high-quality sources with reliable citation tracking, this limited search strategy may have excluded the relevant literature in other databases. Additionally, studies published before 2005 were excluded; although this may omit relevant work, the restriction ensured inclusion of the most current evidence. Given the current review focuses on U.S. college student FI as a representative model, future reviews may expand beyond the U.S. to include college populations worldwide and consider cross-country comparisons across varying levels of economic development.

## 5. Conclusions

Current evidence indicates that FI is not only highly prevalent but also appears to be trending upward in the college population, with disproportionate effects observed among students of color, students from lower socioeconomic backgrounds, and among female students. This review used U.S. college students as a representative population and proposes that current tools be modified to better assess FI in college students, not only nationally, but also globally. Assessment tools should include parameters such as living arrangements, housing and budgeting constraints, parental socioeconomic status, campus food access, and semester-based fluctuations in access to food. These assessment tools should also distinguish between voluntary and access-driven meal skipping. Future studies should also ensure balanced recruitment across gender, race, and socioeconomic status, and incorporate longitudinal designs to enable follow-up and capture changes in FI over time. Together with a more tailored survey instrument, researchers will be able to identify and address college FI, an essential first step before effective interventions can be implemented.

## Figures and Tables

**Table 1:** Summary of food insecurity measurement tools and reported prevalence in studies reviewed.

Measurement Tool(s)	Number of Questions	Target Population	Administration	Key Notes	Studies	Sample Size by Author	FI Rate by Author (%)	FI Range (%)
USDA HFSSM	18	Adults ≥ 12 years	Self-administered	Full FI assessment	*n* = 4	Payne-Sturges, 2018 [14]: 237 Wolfson, 2024 [15]: 2538 Spaid & Gillet-Karam, 2018 [16]: 200+ Leung, 2021 [17]: 1508	Payne-Sturges, 2018 [14]: 15 Wolfson, 2024 [15]: 15 Spaid & Gillet-Karam, 2018 [16]: 52 Leung, 2021 [17]: 14.9	14.9–52
USDA AFSSM	10	Adults ≥ 12 years	Self-administered	Short-form adult measure	*n* = 8	Chaparro, 2009 [18]: 410 Batchelder, 2025 [19]: 4140 El Zein, 2019 [20]: 855 El Zein, 2020 [21]: 683 Brescia & Cuite, 2022 [22]: 6823 Wooten, 2019 [18]: 4824 Fiagbor, 2025 [23]: 462 Glick, 2025 [24]: 852	Chaparro, 2009 [25]: 21 Batchelder, 2025 [19]: 22 El Zein, 2019 [20]: 19 El Zein, 2020 [21]: 25.4 Brescia & Cuite, 2022 [22]: 41.15 Wooten, 2019 [18]: 36 Fiagbor, 2025 [23]: 14 Glick, 2025 [24]: 26.7	14–41.15
USDA HFSSM—Short Form	6	Adults ≥ 12 years	Self-administered	Limited sensitivity for severe FI	*n* = 14	Patton-Lopez, 2014 [26]: 354 Marmolejo, 2024 [27]: 48,103 Martinez, 2019 [28]: 8705 Becerra & Becerra, 2020 [29]: 302 Reeder, 2020 [30]: 131 Tripathy, 2024 [31]: 418 Harville, 2023 [32]: 1087 Mobley, 2024 [33]: 372 Fortin, 2021 [34]: 30 Mei, 2021 [35]: 1033 Raskind, 2019 [36]: 2377 Davidson, 2020 [37]: 943 Zuo, 2026. [38]: 335 Cuy Castellanos, 2020 [39]: 560	Patton-Lopez, 2014 [26]: 59 Marmolejo, 2024 [27]: 40.7 Martinez, 2019 [28]: 40 Becerra & Becerra, 2020 [29]: 37.5 Reeder, 2020 [30]: 32.8 Tripathy, 2024 [31]: 32 Harville, 2023 [32]: 36 Mobley, 2024 [33]: 23.7 Fortin, 2021 [34]: 23 Mei, 2021 [35]: 14 Raskind, 2019 [36]: 29 Davidson, 2020 [37]: 25 Zuo, 2026 [38]: 61 Cuy Castellanos, 2020 [39]: 35.8	14–61
HVSS	2	Primarily Children	Self-administered	Brief screening instrument	*n* = 4	Bruening, 2016 [40]: 209 Zickgraf, 2022 [41]: 121,627 Duke, 2023 [42]: 351 Enriquez & Ader, 2024 [43]: 3155	Bruening, 2016 [40]: 37 Zickgraf, 2022 [41]: 31.9 Duke, 2023 [42]: 72.9 Enriquez & Ader, 2024 [43]: 54.5	31.9–72.9

**Table 2:** Needs for college-specific adaptation of FI measurement tools.

Measurement Tool(s)	Identified Needs in College Populations
USDA HFSSM (Full Scale)	Adaptation to student living arrangements (e.g., shared housing, parental support, independent budgeting) Inclusion of campus-based food access (e.g., meal plans, dining halls) Better capture of temporary vs. chronic FI (e.g., semester-based fluctuations)
USDA HFSSM (Short Form)	Expanded items to assess severity and frequency College-specific follow-up questions (context of when/why insecurity occurs) Improved differentiation between voluntary meal skipping and resource limitation Adjustment for non-traditional food access systems (e.g., campus dining)
AFSSM	Incorporation of student financial dependency contexts Consideration of academic calendar effects on food access Increased sensitivity to mild and moderate FI common in students
HVSS(;)	Addition of follow-up questions to assess severity Expansion beyond binary screening to include frequency and duration Student-specific framing (e.g., within-semester reference period) Optional supplementary module capturing risk factors (housing, campus access, financial support)

## Data Availability

No new data were created or analyzed in this study. Data supporting the findings are available within the published literature cited in this review.

## Supplementary Materials

The following supporting information can be downloaded at https://www.mdpi.com/article/10.3390/nu18121866/s1, PRISMA 2020 Checklist. Table S1: JBI Checklist. Reference [55] is cited in the supplementary materials.

## Abbreviations

The following abbreviations are used in this manuscript:

FI	Food Insecurity
USDA	United States Department of Agriculture
HFSSM	Household Food Security Survey Module
AFSSM	Adult Food Security Survey Module
HVSS	Health Watch Vital Sign
PRISMA	Preferred Reporting Items for Systematic Reviews and Meta-Analysis
OR	Odds Ratios
HBCUs	Historically Black Colleges and Universities
LPSU	Large Public Southeastern University
SNAP	Supplemental Nutrition Assistance Program
WIC	Special Supplemental Nutrition Program for Women, Infants, and Children
AAU	Association of American Universities
